# Bilateral ocular ischemia-induced blindness as a presenting manifestation of Takayasu arteritis: a case report

**DOI:** 10.1186/s13256-017-1330-3

**Published:** 2017-06-10

**Authors:** Pedro Pallangyo, Emmanuel Epafra, Paulina Nicholaus, Frederick Lyimo, Parvina Kazahura, Mohamed Janabi

**Affiliations:** 1 Department of Cardiovascular Medicine, Jakaya Kikwete Cardiac Institute, P.O. Box 65141, Dar es Salaam, Tanzania; 2 Department of Radiology, Muhimbili National Hospital, P.O. Box 65000, Dar es Salaam, Tanzania

**Keywords:** Takayasu arteritis, Occlusive thromboaortopathy, Granulomatous panarteritis, Chronic granulomatous vasculitis, Large vessel vasculitis, Pulseless disease, Case report

## Abstract

**Background:**

Takayasu arteritis is a granulomatous panarteritis that predominantly affects the aorta and its major branches. The initial manifestations of this large-vessel vasculitis are usually nonspecific; however, as the disease progresses, typical symptoms of arterial occlusion, aneurysmal formation, and vascular pain become evident. Ischemic ocular complications of Takayasu arteritis which could lead to complete loss of vision are not uncommon and depend on the obliterated portion(s) of carotid(s), the intensity and rate of progression of ocular vascular insufficiency, and sufficiency of the collateral blood supply to the eye.

**Case presentation:**

A 24-year-old woman of African descent with prior normal vision was referred to us with a 3-year history of gradual decline in visual acuity in both eyes and unintentional weight loss (17 kg) within the past 1 year. A physical examination revealed feeble brachial and radial arterial pulses on her left side. She had sinus tachycardia (136 beats/minute) and her blood pressure was 85/59 mmHg on her left and 134/82 mmHg on her right side. Bilateral microaneurysms, dot and blot hemorrhages, and multiple ischemic areas of retina together with neovascularization in her right eye were noted during a funduscopic examination. Computed tomography angiography of her thoracic and abdominal aorta revealed irregular narrowing with variable degrees of stenosis, tapering, and corrugated appearance.

**Conclusions:**

Despite its rarity, Takayasu arteritis significantly impairs a patient’s quality of life and has a life-threatening potential. Early initiation of appropriate therapy could delay disease progression and reduce the associated complications.

## Background

Takayasu arteritis (TA) is a rare, idiopathic, autoimmune, and progressive chronic granulomatous vasculitis that is largely (90%) seen in young women (<40 years) [1–7]. This nonspecific aortoarteritis predominantly affects the aorta and its major branches; however, coronary, renal, and pulmonary arteries involvement has been documented [1–3]. The diffuse nature of TA leads to multiorgan system involvement with varying degrees of pathological features including thickening, fibrosis, stenosis, aneurysm, and thrombus formation in involved portions of arteries [3, 8, 9].

The initial stage (pre-vasculitic phase) of TA lacks a diagnostic test as it manifests with nonspecific symptoms including malaise, fever, headache, arthralgias, and weight loss [10–12]. During the late stage (pulseless phase), symptoms of arterial occlusion, aneurysmal formation, and vascular pain (carotidynia) are evident and patients present with a myriad of clinical syndromes like pulseless extremities, claudication, vascular bruits, hypertension, Raynaud’s syndrome, pericarditis, aortic regurgitation, congestive heart failure, stroke, and myocardial infarction [10–12]. Erythema nodosum is the commonest dermatologic manifestation of TA; other dermatologic manifestations, including ulcerated subacute nodular lesions and pyoderma gangrenosum, occur less frequently [11, 12]. Neurological manifestations including seizures, syncope, blindness, and stroke are not uncommon and may be the initial presentation of the disease [8, 11–13]. We report a case of blindness as the initial presentation of TA in a 24-year-old woman from Tanzania.

## Case presentation

A 24-year-old woman of African descent with prior normal vision was referred to us from a specialized ophthalmic center for a preoperative cardiovascular assessment. She had chief complaints of gradual decline in visual acuity in both eyes for 3 years and unintentional weight loss (17 kg) within the past 1 year. Her past medical history was unremarkable except for a self-reported history of pulmonary tuberculosis at the age of 4 years. Her progressive loss of vision which initially started in her left eye and later involved her right eye was associated with intermittent pain, redness, tearing, and headaches. Within the first year, she completely lost vision in her left eye. She denied any history of arthralgias, claudication, skin lesions, chest pain, convulsions, or syncope. She had several out-patient visits in the past 3 years all due to her ongoing visual impairment. She was prescribed gentamycin eye drops, carbimazole, propranolol, furosemide, and multivitamin capsules at different points in time with no relief of her deteriorating vision.

Her physical examination revealed feeble brachial and radial arterial pulses on her left side, whereas pulses on her right side and in both lower limbs were normal. Her blood pressure was 85/59 mmHg on her left and 134/82 mmHg on her right side. She had a pulse rate of 136 beats/minute which was regular and of good volume, body temperature of 37.0 °C, and a body mass index (BMI) of 19.6 kg/m^2^. On ophthalmic examination, she had no light perception in her left eye but she could count fingers at a 2-meter distance using her right eye. Bilateral microaneurysms, dot and blot hemorrhages, and multiple ischemic areas of retina together with neovascularization in her right eye were noted during a funduscopic examination. These ophthalmic findings are in line with a bilateral ocular ischemic syndrome.

**Fig. 1:**
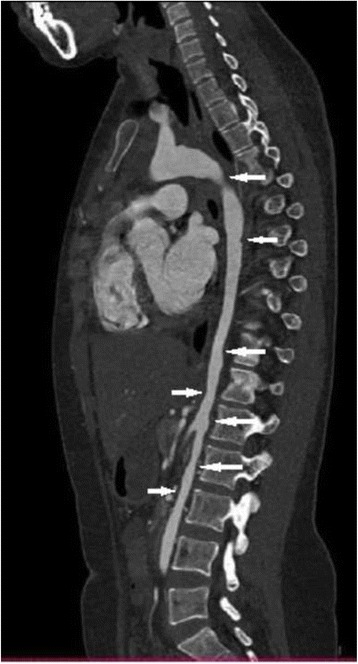
Computed tomography angiography of the thoracic and abdominal aorta showing irregular narrowing with variable degrees of stenosis (*arrows*) and tapering

**Fig. 2:**
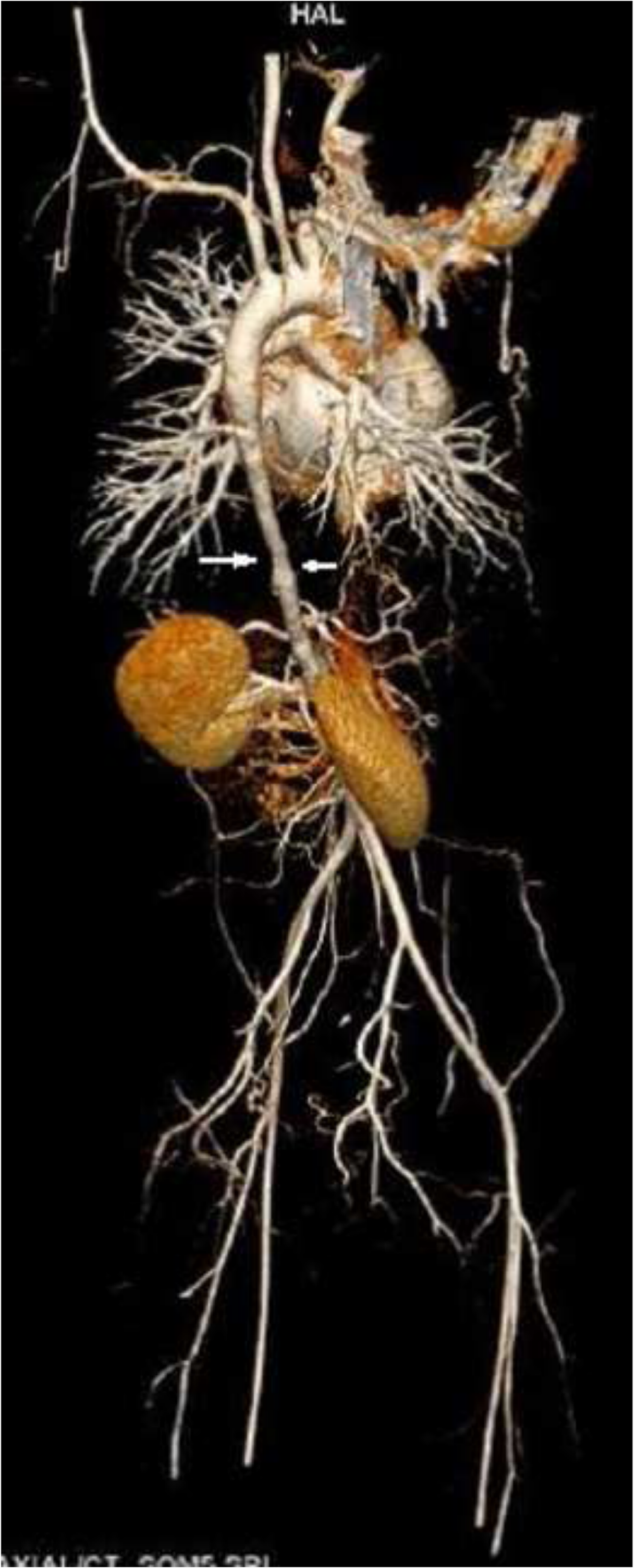
Three-dimensional computed tomography scan of the thoracic and abdominal aorta showing irregular narrowing with variable degrees of stenosis (*arrows*), tapering and corrugated appearance

**Fig. 3:**
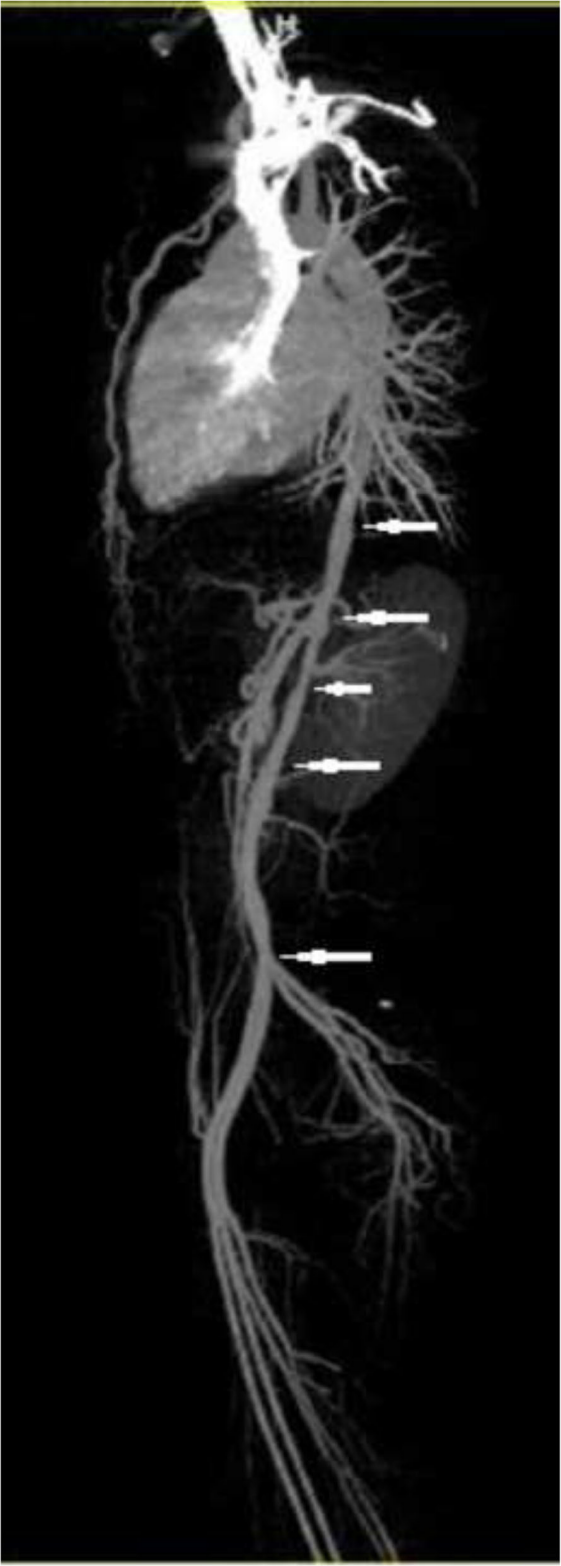
Computed tomography angiography of the thoracic and abdominal aorta showing irregular narrowing with variable degrees of stenosis (*arrows*), tapering and corrugated appearance

Laboratory results revealed an elevated erythrocyte sedimentation rate (ESR) and C-reactive protein (CRP) of 56 mm/hour and 25.4 mg/L respectively. Electrolytes, liver, renal, and thyroid function tests were normal. Serology for human immunodeficiency virus (HIV), hepatitis B and C, Venereal Disease Research Laboratory (VDRL) test for syphilis, rheumatoid factor, anti-double-stranded DNA (dsDNA), and antineutrophil cytoplasmic antibody (ANCA) were negative, however, antinuclear antibody (ANA) was positive. A tuberculin test was negative. There were no aneurysmal formations, bruits, ventricular hypertrophy, or any other cardiac abnormalities apart from a sinus tachycardia and trace aortic regurgitation. We did not perform coronary angiography or computed tomography (CT) angiography of her head and neck due to her financial constraints. A CT angiogram of her thoracic and abdominal aorta revealed irregular narrowing with variable degrees of stenosis, tapering, and corrugated appearance (Figs. 1, 2, and 3). Based on the clinical presentation, physical findings, and angiographic features, she fulfilled the American College of Rheumatology criteria [8] for Takayasu’s arteritis. However, we could not rule out temporal arteritis in this case as our patient refused a temporal artery biopsy procedure. Nevertheless, based on the age of onset (that is, <40 years) and the clinicoradiographic picture (decreased pulsation of brachial artery, difference of over 10 mmHg in systolic blood pressure between arms, and arteriographic narrowing of aorta) we entertained a diagnosis of TA. She was counseled and treated with dexamethasone (60 mg per day), methotrexate (10 mg per day), and low-dose aspirin (75 mg once daily). She was discharged home on self-request after 9 days of hospitalization. We reviewed her as an out-patient after 3 months but there was neither improvement nor further deterioration in her visual acuity despite self-reported good adherence.

## Discussion

TA is a nonspecific granulomatous polyarteritis with a poorly understood pathogenesis. This systemic vasculopathy has a variable course ranging from a rapid progression in some patients to a late quiescent phase in others [14]. In up to 90% of cases there is involvement of the aorta, subclavian arteries, and carotid arteries, and angiographic features including stenosis, occlusion, dilatation, or aneurysm are pathognomonic [3, 15]. Diagnosis of TA is a challenge to practitioners worldwide largely due to its nonspecific presentation, multiorgan system involvement, and lack of diagnostic serologic tests. For instance, ESR elevation is a common finding but nearly half of patients with active disease have a normal sedimentation rate [13]. The presence of at least three of six characteristics according to the American College of Rheumatology criteria for TA is diagnostic [13]. In the case presented, four criteria including age ≤40 years, decreased brachial artery pulse, greater than 10 mmHg difference in systolic blood pressure between arms, and arteriographic evidence of narrowing of the aorta were present.

Management of TA aims at suppressing the inflammatory process and preservation of vascular capability. Corticosteroids (that is, prednisolone) and methotrexate are a commonly used anti-inflammatory therapy while the addition of low-dose aspirin is also advocated. Approximately 20% of patients with TA are resistant to any therapy and despite good adherence to immunosuppressive medications, remission occurs only in approximately half of cases [13]. Endovascular revascularization procedures including stent placement, bypass grafts, endarterectomy, and angioplasty should be considered in patients with symptomatic stenosis or occlusion [1]. Although such surgical and endovascular interventions have a potential for short-term benefits, failure and poorer outcomes, especially when they are undertaken during active inflammatory phase, are often reported [1]. Overall, the prognosis of TA is good with reported 5-year survival rates of 90% [13–15].

The diagnosis of TA in the case presented was reached after ocular ischemia-induced blindness had set in. Ocular ischemic syndrome is a hypoperfusive retinopathy resulting from chronic hypoperfusion of the ophthalmic artery secondary to carotid artery stenosis [16–20]. The ischemic ocular complications of TA which could lead to complete loss of vision depend on the obliterated portion(s) of carotid(s), the intensity and rate of progression of ocular vascular insufficiency, and sufficiency of the collateral blood supply to the eye [18]. This case demonstrates the enigmatic character of TA and echoes the importance of a high index of suspicion for early diagnosis.

## Conclusions

In conclusion, despite its rarity, TA significantly impairs a patient’s quality of life and has a life-threatening potential. Early initiation of appropriate therapy could delay disease progression and reduce the associated complications.

## Abbreviations

ANA: Antinuclear antibody
ANCA: Antineutrophil cytoplasmic antibody
BMI: Body mass index
CRP: C-reactive protein
CT: Computed tomography
dsDNA: Double-stranded deoxyribonucleic acid
ESR: Erythrocyte sedimentation rate
HIV: Human immunodeficiency virus
TA: Takayasu arteritis
VDRL: Venereal Disease Research Laboratory
